# Anti-Adhesion Effects and Potential Mechanisms of Polyglutamic Acid–Polylysine Crosslinked Hydrogels in a Rat Peritoneal Adhesion Model

**DOI:** 10.3390/gels12090857

**Published:** 2026-09-21

**Authors:** Shize Wu, Zixi Zhao, Donghe Jia, Huiying Li, Yijing Huang, Qianqian Han

**Affiliations:** 1 National Institutes for Drug Control, Beijing 102629, China; 13623044404@163.com (S.W.); zhaozixi@nifdc.org.cn (Z.Z.); jdh2501191477@163.com (D.J.); lihuiying440@163.com (H.L.); huangyj0129@163.com (Y.H.); 2 Department of Pharmaceutics, China Pharmaceutical University, Nanjing 211198, China; 3 School of Medical Devices, Shenyang Pharmaceutical University, Benxi 117004, China

**Keywords:** peritoneal adhesions, hydrogel, polyglutamic acid, polylysine, anti-fibrosis, inflammation

## Abstract

Peritoneal adhesions are a common complication following abdominal surgery. Their formation is associated with factors such as inflammatory responses, imbalance in the fibrinolytic system, and proliferation of fibrous tissue. Currently available anti-adhesion products on the market are primarily physical barrier membranes, which have limited functionality, can only serve as passive barriers, and have suboptimal handling and residence times. To address this issue, this study utilized 1-ethyl-3-(3-dimethylaminopropyl)carbodiimide hydrochloride/N-hydroxysuccinimide (EDC/NHS) chemical crosslinking to create polyglutamic acid-polylysine (PGA-PL) hydrogels and tested their anti-adhesion and reparative effects in a cecum–abdominal wall adhesion model in Sprague–Dawley (SD) rats. Experimental results demonstrated that treatment with PGA-PL hydrogels significantly reduced the occurrence of adhesions, effectively decreased inflammatory cell infiltration, neovascularization, and collagen deposition, while exhibiting excellent biocompatibility. Additionally, it markedly reduced the expression of pro-inflammatory factors, transforming growth factor-β1 (TGF-β1), and α-smooth muscle actin (α-SMA) in both serum and local tissue, increased the levels of plasminogen activators, and promoted the proliferation of peritoneal mesothelial cells. PGA-PL hydrogel is a safe and effective anti-adhesion material; its efficacy not only relies on serving as a physical barrier but also actively modulates local inflammatory responses, fibrotic processes, and fibrinolytic balance, while promoting mesothelial repair.

## 1. Introduction

The reported incidence of postoperative peritoneal adhesions varies between 55% and 66%, which renders it a leading cause of multiple postoperative complications including intestinal obstruction, chronic abdominal pain, and secondary infertility [1,2,3]. The formation of adhesions is a complex pathological process involving imbalances in inflammation, coagulation, fibrosis, and tissue repair. The formation of postoperative peritoneal adhesions begins with mechanical injury, ischemia, and inflammatory stimulation of peritoneal mesothelial cells during surgery, thereby initiating a complex cascade of events involving three core components: the inflammatory response, an imbalance in the fibrinolytic system, and fibrous tissue proliferation [4]. Damaged peritoneal mesothelial cells, along with aggregated platelets, neutrophils, and macrophages, release large amounts of inflammatory mediators and cytokines [5,6,7]. Among these, tumor necrosis factor-α (TNF-α) and interleukin-6 (IL-6), as key early pro-inflammatory factors, not only amplify the inflammatory cascade but also promote chronic inflammation through positive feedback loops (e.g., IL-6 signaling stimulates neutrophils to produce TNF-α) [8,9,10]. Concurrently, transforming growth factor-β1 (TGF-β1), as a central pro-fibrotic factor, induces mesothelial-mesenchymal transition (MMT) in peritoneal mesothelial cells by activating classical Smad and non-classical MAPK/PI3K signaling pathways, thereby transforming them into secretory myofibroblasts [11,12]. Under physiological conditions, tissue-type plasminogen activator (t-PA) converts plasminogen into active plasmin to mediate fibrin degradation. However, after peritoneal injury, the synthesis and secretion of t-PA by mesothelial cells are markedly downregulated, while the expression of its specific endogenous inhibitor, plasminogen activator inhibitor-1 (PAI-1), is significantly elevated, resulting in impaired fibrinolytic function and insufficient fibrin clearance [13]. This, combined with increased vascular permeability and the leakage of fibrinogen, leads to excessive deposition of fibrin on the damaged peritoneal surface, forming a temporary matrix [14]. If not promptly cleared, this fibrin matrix, under continuous stimulation by factors such as TGF-β1, serves as a scaffold for the infiltration and proliferation of MMT-associated cells and activated fibroblasts [15]. Concurrently, pathological angiogenesis mediated by factors such as vascular endothelial growth factor (VEGF) provides nutritional support for the maturation of the adhesion band, ultimately leading to the formation of permanent fibrous adhesions rich in blood vessels and collagen [16].

Although precise surgical techniques form the foundation of prevention, the clinical use of anti-adhesion materials is often necessary. Current mainstream products, such as absorbable barrier membranes and gels based on hyaluronic acid or polyethylene glycol, primarily provide passive physical separation [17,18]. However, they remain limited in terms of retention in the moist peritoneal environment, tissue adhesion, and conformability to complex wound surfaces. Furthermore, most materials are functionally limited and lack the ability to actively modulate the microenvironment of the injury site [19]. Therefore, the development of novel materials that combine ideal barrier functions with active biological activity is a current research focus. γ-polyglutamic acid is a natural anionic peptide with good biocompatibility, moisture retention, and modifiability [20]. ε-polylysine is a natural cationic peptide with broad-spectrum antimicrobial activity. The two can form composite hydrogels through electrostatic interactions and chemical crosslinking [21]. We hypothesize that hydrogels constructed from these two bioactive peptides not only form a physical barrier but that their components or degradation products may also exert active anti-adhesion effects by modulating immune responses and influencing the fibrosis process.

In this study, PGA-PL hydrogels were prepared using the EDC/NHS crosslinking method. Their physicochemical properties were systematically evaluated, and their efficacy in preventing postoperative peritoneal adhesions was comprehensively investigated at multiple levels—including overall therapeutic efficacy, systemic safety, and molecular mechanisms—using a rat peritoneal adhesion model. The aim is to comprehensively evaluate their anti-adhesion efficacy and preliminarily explore the potential underlying mechanisms.

## 2. Results and Discussion

### 2.1. Antifouling Properties of PGA-PL Hydrogels

**Figure 1:**
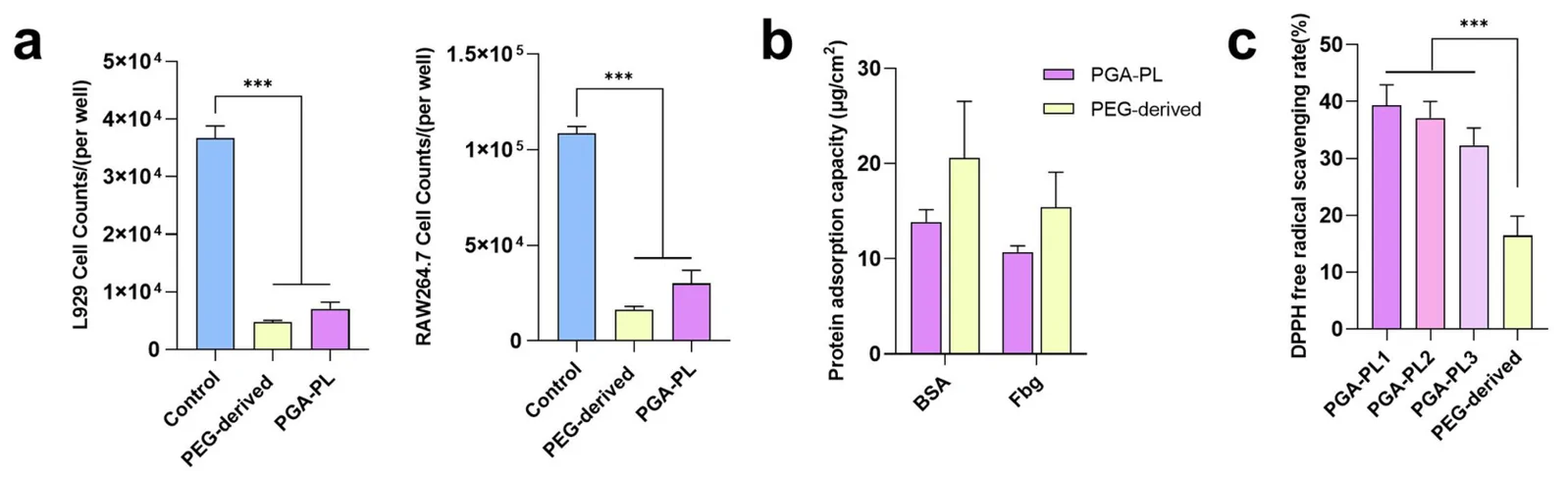
(**a**) Number of L929 and RAW 264.7 cells adhering to TCPS, PGA-PL, and PEG-derived hydrogels (*n* = 3); (**b**) Adsorption capacity of PGA-PL and PEG-derived hydrogels for proteins (BSA, Fbg) (*n* = 3); (**c**) Quantitative analysis of the DPPH radical scavenging efficiency of PGA-PL hydrogels (*n* = 4). *** *p* < 0.001.

**Figure A1:**
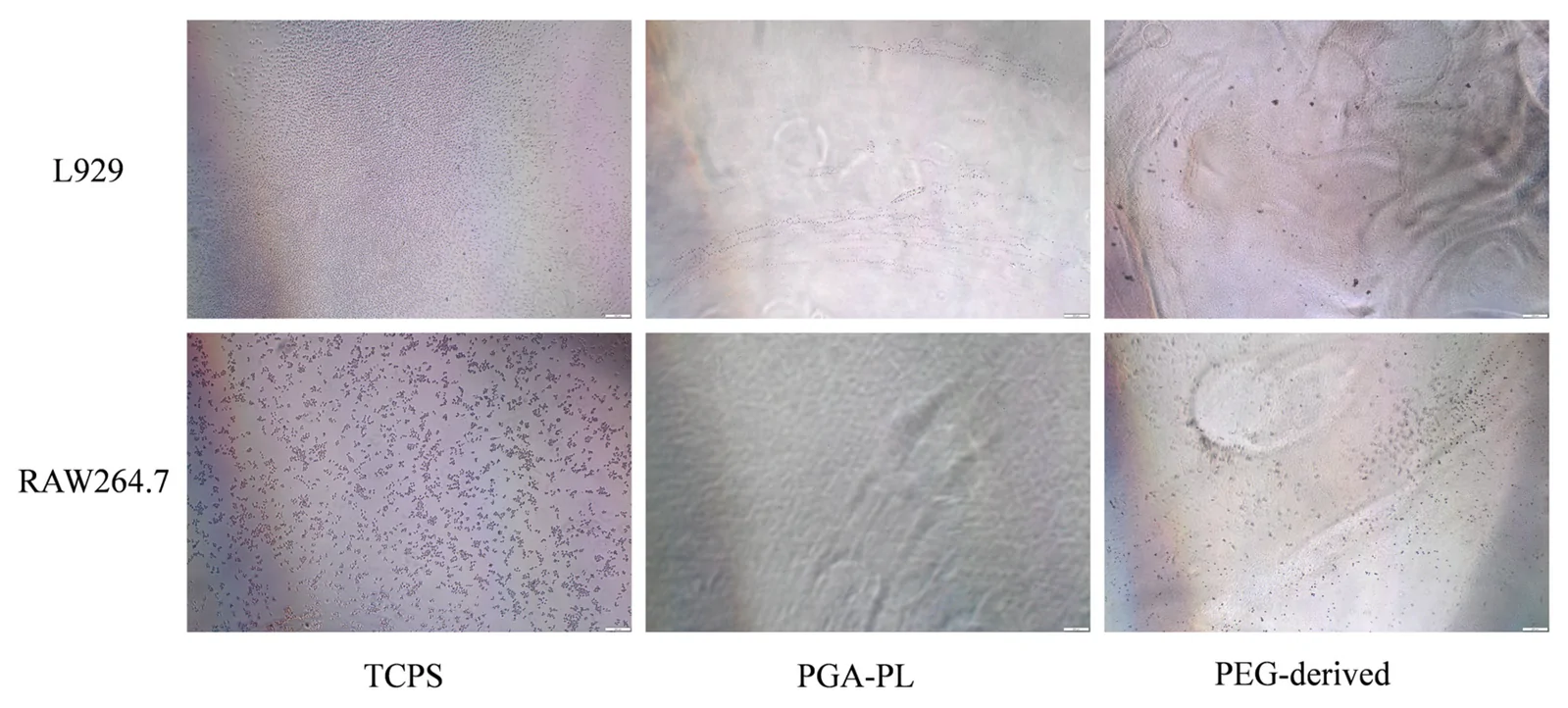
Adhesion of L929 and RAW 264.7 cells to TCPS, PGA-PL, and PEG-derived hydrogels (scale bar: 200 μm).

Non-specific protein adsorption and cell adhesion are inevitable challenges during the implantation of hydrogels into the peritoneal cavity, and excess collagen, fibroblasts, and macrophages are direct contributors to the formation of adhesions. Therefore, a comprehensive evaluation of the anti-fouling capabilities of CGM hydrogels is crucial. After 24 h of incubation with L929 and RAW264.7 cells, imaging of the hydrogel and TCPS surfaces revealed that a large number of morphologically normal L929 and RAW264.7 cells were present on the TCPS, whereas the PGA-PL hydrogel and commercial PEG-derived hydrogel surfaces exhibited very few cells, and those present displayed abnormal morphology (Figure A1). Cell counting was performed for each group, revealing that compared to the control group, the number of adherent fibroblasts and macrophages in each hydrogel group was significantly reduced (Figure 1a). These cells can secrete charged proteins to enhance substrate adhesion; however, both PGA and PL are strongly hydrophilic polyelectrolytes, and when they form hydrogels through electrostatic interactions, their surfaces typically exhibit high hydrophilicity and strong charge. This may form a tight hydration layer that actually prevents adhesion proteins on the cell membrane from approaching the hydrogel surface.

To further verify the anti-fouling capacity of PGA-PL hydrogel compared with commercially available anti-adhesive hydrogels, bovine serum albumin (BSA) and fibrinogen (Fbg) were used to investigate its resistance to protein adsorption. The results showed that the adsorption amounts of both proteins on PGA-PL hydrogel were lower than those on PEG-based hydrogel, indicating its superior performance in resisting non-specific protein adsorption.

### 2.2. DPPH Free Radical Scavenging Capacity of PGA-PL Hydrogels

The abundant carboxyl groups in PGA and the abundant amino groups in PL are the primary active sites for DPPH scavenging. However, as the degree of crosslinking increases, more active groups are consumed in the reaction, resulting in fewer sites available for hydrogen donation and radical quenching. Figure 1c shows the DPPH radical scavenging rates for the high, medium, and low PGA-PL groups and the PEG-derived group. It was found that as the PL ratio decreased, the DPPH scavenging rate of the PGA-PL groups showed a slight downward trend, but their scavenging capacity was still significantly superior to that of commercial PEG hydrogels. This phenomenon can be explained by the synergistic antioxidant effect between the amine groups on PL side chains and the carboxyl groups on PGA chains. The incorporation of PL not only participates in crosslinking to construct the three-dimensional hydrogel network, but also provides additional amine groups that can serve as electron donors to scavenge DPPH radicals. Furthermore, higher PL content brings more reactive sites into the hydrogel system, thus leading to stronger radical scavenging efficacy. In contrast, PEG-derived hydrogels lack functional groups that can directly participate in radical quenching reactions, resulting in relatively weak DPPH scavenging performance.

These results indicate that PGA-PL hydrogels, while possessing anti-adhesion properties, also provide significant antioxidant activity, offering potential advantages in postoperative anti-adhesion dressings.

### 2.3. Effect of PGA-PL Hydrogel in a Cecum–Peritoneum Adhesion Model

All 40 rats survived the surgery. Since the peritoneum heals within 5–7 days regardless of the extent of the peritoneal injury, the occurrence of adhesions also depends on the period between 5 and 7 days post-surgery. Therefore, we chose to observe for adhesions at 7 days and further verify the results and progression of adhesions at 14 days.

At 7 days after surgery, all 5 rats in the model control group developed visible peritoneal adhesions. In the commercial PEG-derived hydrogel treatment group, 1 rat presented moderate adhesions while the other 4 showed no detectable adhesions. For the PGA-PL hydrogel group, 4 animals exhibited no adhesion formation and only 1 showed mild adhesion. At 14 days post-surgery, all 5 animals in the model control group still presented distinct adhesions. In the commercial PEG-derived hydrogel group, 1 rat showed severe adhesions, 3 had mild adhesions, and only 1 was free of adhesions. In contrast, none of the 5 ceca in the PGA-PL hydrogel group showed adhesion to the abdominal wall; only one specimen presented a single thin adhesion band between the lower abdominal wall and surrounding adipose tissue. The blank control group received no surgical trauma, so their adhesion scores were 0 at both day 7 and day 14. Detailed adhesion scoring results for each group are summarized in Table A1.

**Figure 2:**
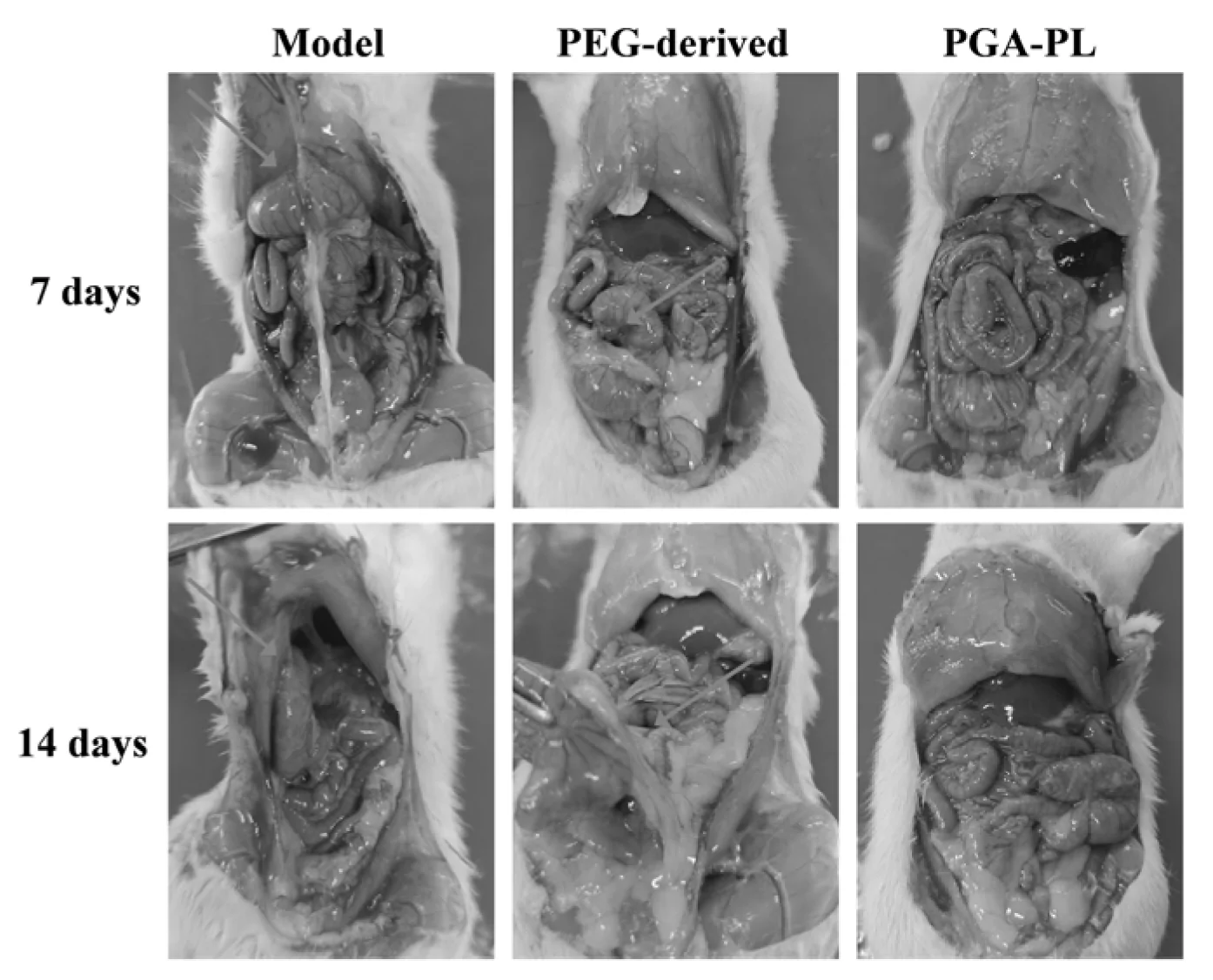

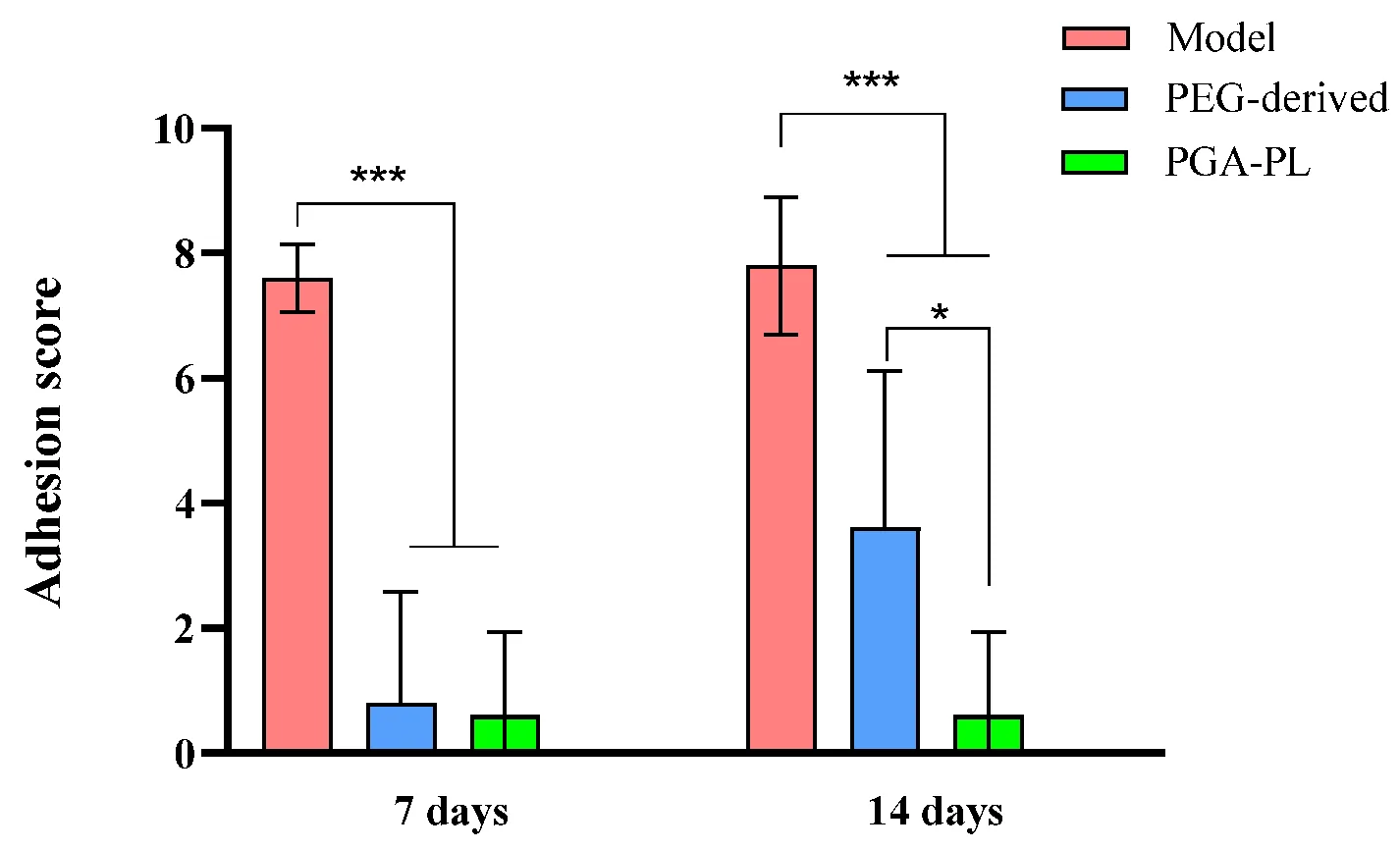
Gross evaluation of postoperative adhesions and adhesion scores for each group at 7 and 14 days postoperatively (*n* = 5). * *p* < 0.05, *** *p* < 0.001.

As shown in Figure 2, at day 7 post-surgery, the adhesion scores of both the PGA-PL hydrogel group and the PEG-derived hydrogel group were significantly lower than that of the model control group (*p* < 0.001), and no statistically significant difference was observed between the two treatment groups. Further analysis of day 14 results revealed that compared with the model control group, the adhesion score of the commercial PEG-derived hydrogel group was also significantly reduced (*p* < 0.001), but it was still significantly higher than that of the PGA-PL hydrogel group (*p* < 0.05).

The adhesion scores indicate that this PGA-PL hydrogel provides good postoperative anti-adhesion effects and is slightly superior to commercially available PEG-derived hydrogels.

### 2.4. Histological Evaluation

**Figure 3:**
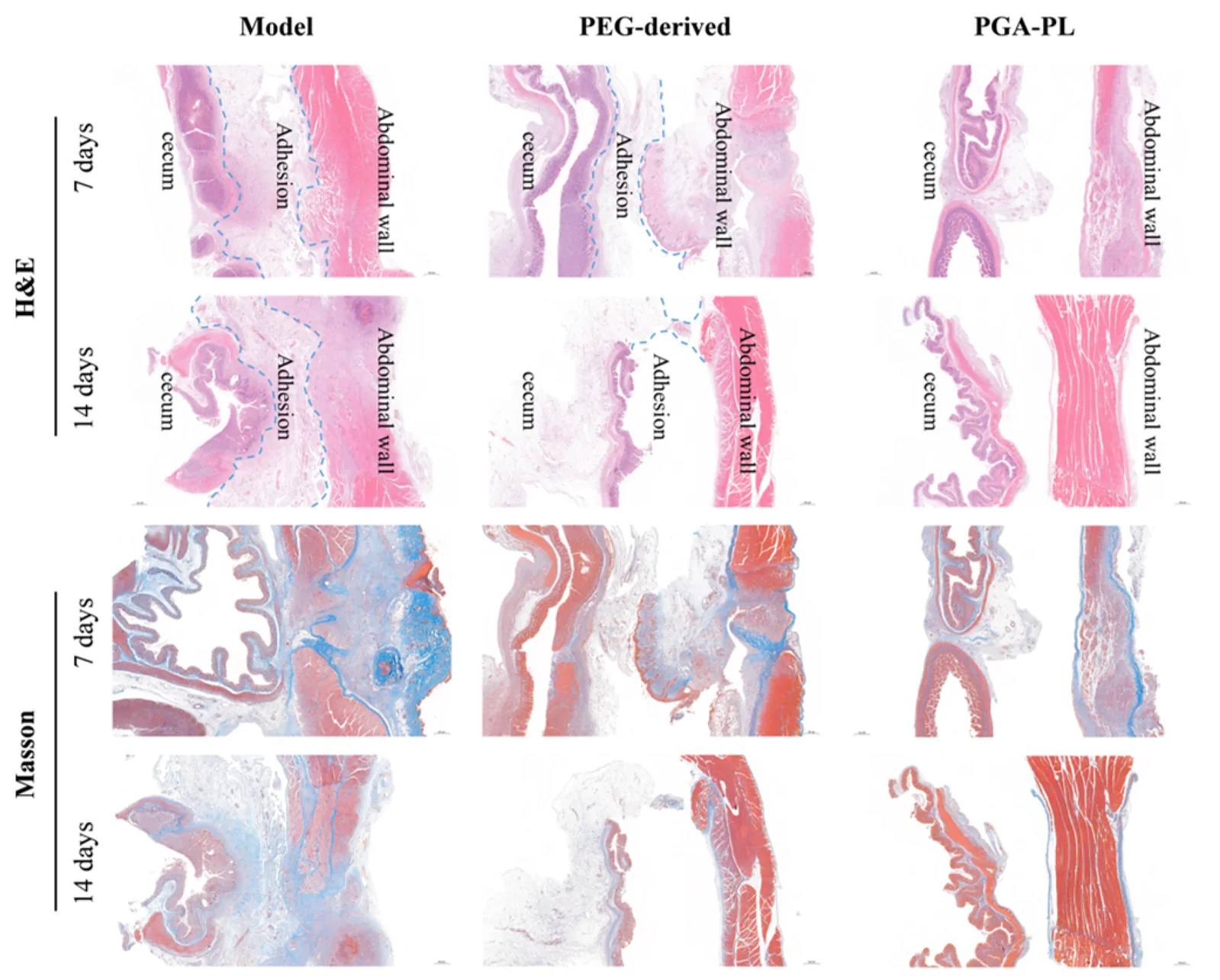
H&E and Masson’s stain results for the area of localized adhesions.

H&E staining and Masson staining were used for histological examination of the local adhesion bands. As shown in Figure 3, H&E staining of the control group in the 7-day and 14-day models revealed that the visceral and parietal peritoneum had completely fused into adhesion bands. Within these bands, infiltration of inflammatory cells (lymphocytes, neutrophils, etc.), neovascularization, and extensive fibroblast proliferation were observed; Masson staining revealed dense, thick deposits of collagen fibers. Following treatment with the commercial PEG-derived hydrogel, adhesion conditions improved in most rats; however, some adhesion bands were not fully resolved. Although the thickness of the adhesion bands decreased, inflammatory cells and a small amount of neovascularization were still observed; Masson staining also showed collagen fiber deposits in this group. Compared with the control and commercial groups, no adhesion bands were observed in the PGA-PL group at either 7 or 14 days post-surgery. H&E staining revealed that by day 14, the parietal peritoneum had completely healed and was covered with new mesothelial cells, with no inflammatory response; Masson staining results further showed a significant reduction in collagen fiber content within the peritoneum.

Taken together, the H&E and Masson’s staining results indicate that PGA-PL hydrogel can suppress fibroblast proliferation, inhibit inflammatory cell infiltration and collagen fiber deposition, thus maintaining the structural integrity of the cecum and promoting peritoneal wound healing.

### 2.5. Blood Biochemistry Analysis Results

**Figure 4:**
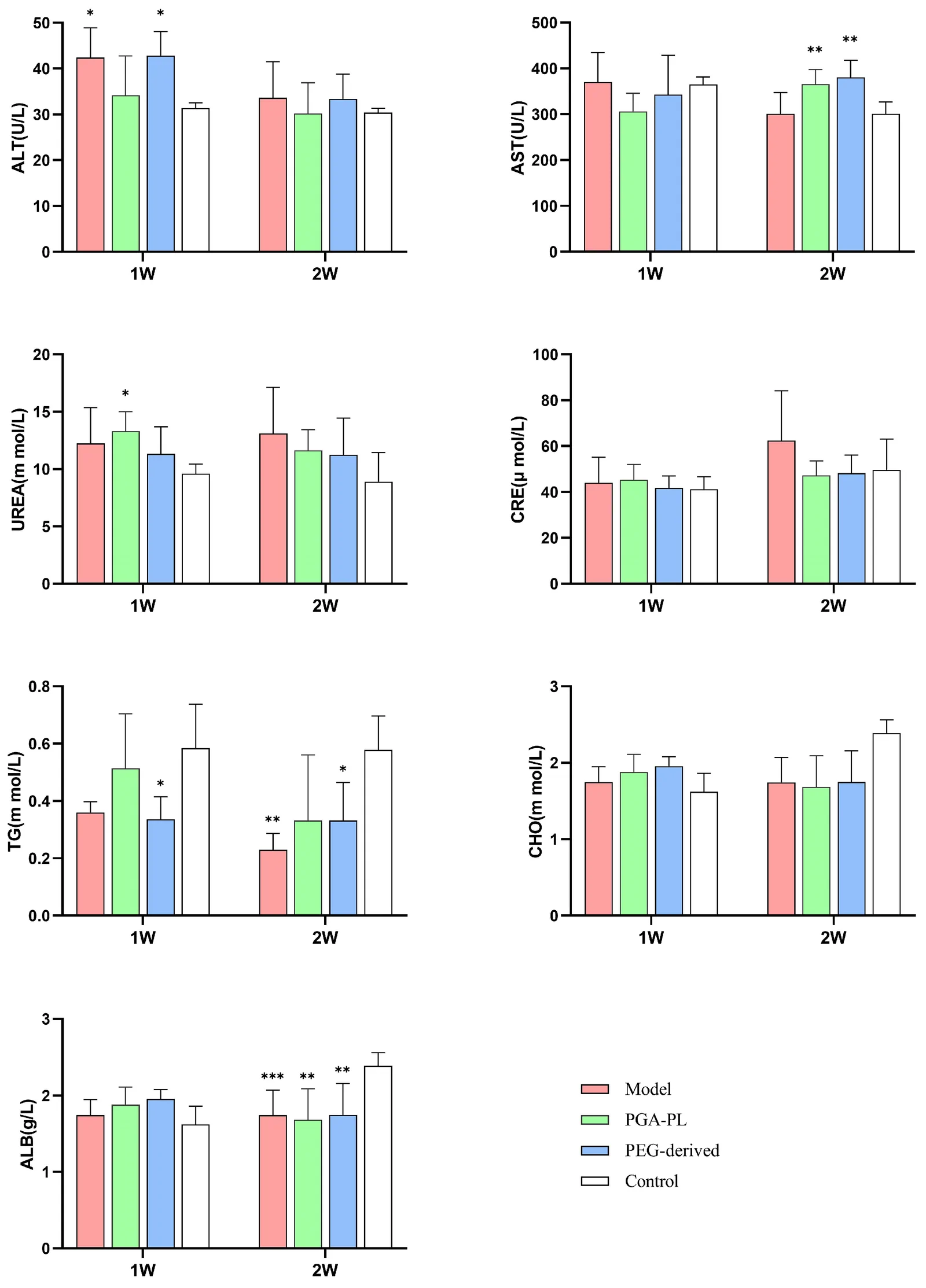
Results of selected blood biochemical tests for rats in each group one and two weeks post-surgery. * *p* < 0.05, ** *p* < 0.01, *** *p* < 0.001.

The blood biochemical test results for rats in each group after surgery are shown in Figure 4.

ALT and AST are sensitive markers of hepatocyte damage. These two enzymes are primarily found within hepatocytes; when the hepatocyte membrane is damaged or undergoes necrosis, they are released into the bloodstream, leading to elevated serum levels. The PEG-derived hydrogel showed a significant increase in AST levels during the second week, suggesting a higher potential risk of hepatotoxicity compared to the PGA-PL group. ALP is widely distributed in tissues such as the liver, bones, and intestines. In the liver, ALP is primarily produced by bile duct epithelial cells. When bile excretion is obstructed, ALP levels rise significantly. ALP levels in all experimental groups showed a downward trend in the second week and exhibited no significant difference compared to the control group. This indicates that neither gel caused biliary obstruction or associated hepatobiliary dysfunction.

Urea and creatinine are waste products of protein metabolism, primarily excreted through glomerular filtration. When renal function is impaired and filtration rate decreases, these substances accumulate in the blood, leading to elevated levels. At all time points, UREA and CRE levels in both gel groups showed no statistically significant increase compared to the control group. This indicates that neither gel caused detectable damage to renal function in SD rats.

TG and CHO are the primary lipids in the blood. Elevated levels may indicate lipid metabolism disorders and are associated with risks such as cardiovascular disease and fatty liver. In the second week, TG levels in both experimental groups were lower than those in the control group, which may reflect changes in the body’s metabolic state but does not constitute an adverse effect. Therefore, neither gel caused harmful dyslipidemia.

Albumin (ALB) is exclusively synthesized by the liver and is the most abundant protein in serum; it plays a crucial role in maintaining plasma osmotic pressure and mediating substance transport. Serum ALB level is a key indicator for evaluating hepatic synthetic function and the nutritional status of the body; decreased ALB level may indicate impaired liver function, malnutrition, or chronic wasting diseases. By the second week post-surgery, the ALB levels in the model control group were significantly lower than those in the blank control group. This finding suggests that abdominal surgical trauma alone is sufficient to keep rats in a chronic wasting state two weeks after surgery, with serum albumin levels failing to return to normal. In both hydrogel treatment groups, especially the PGA-PL group, the ALB levels were slightly higher than those in the model control group, but still significantly lower than the blank control group. This indicates that the reduction in serum albumin was mainly caused by the abdominal surgical trauma itself, rather than an additional side effect of hydrogel implantation. Between the two tested materials, PGA-PL showed better efficacy in alleviating the chronic wasting state induced by surgical trauma.

### 2.6. Pathological Findings in Major Organs

**Figure 5:**
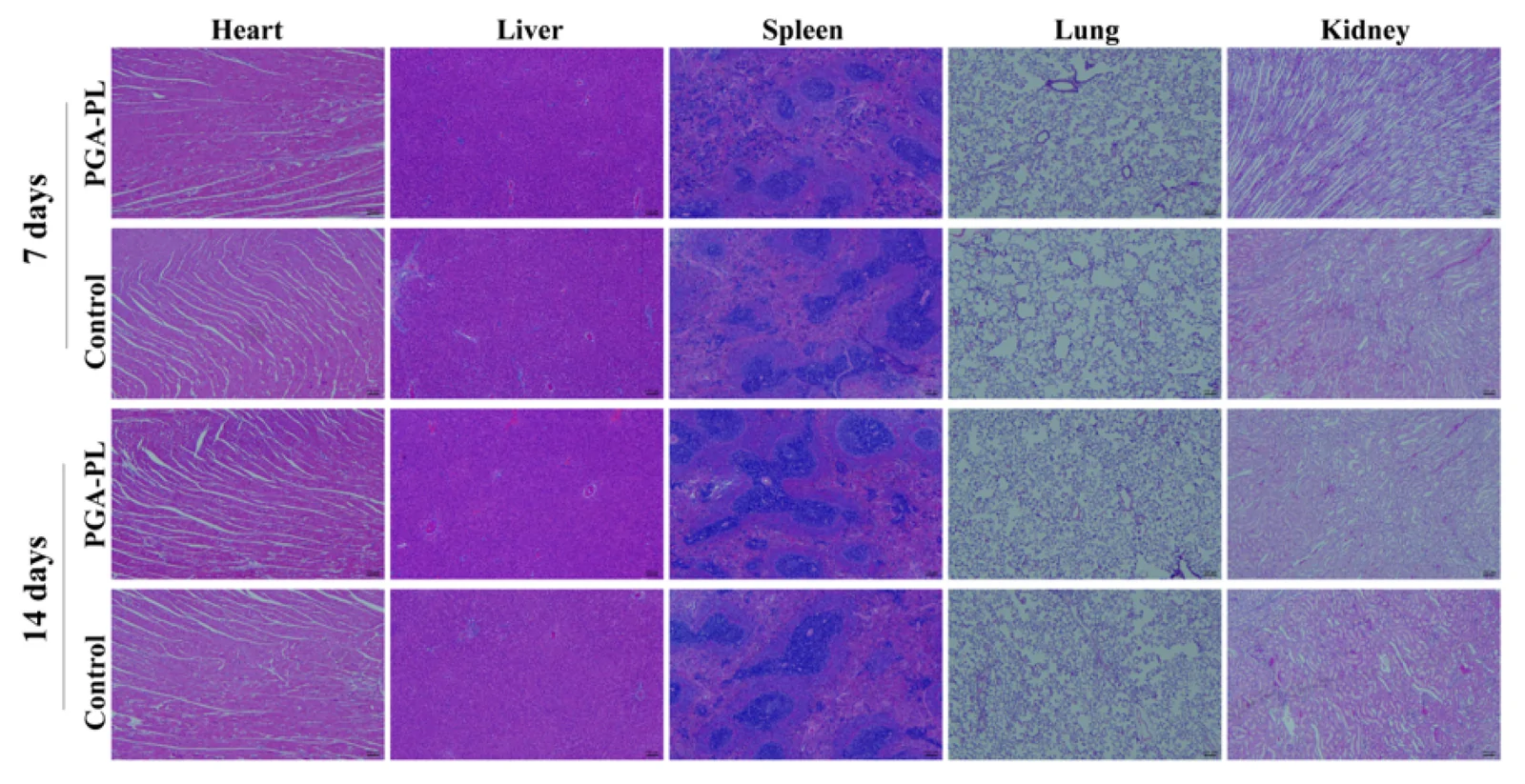
HE staining of major rat organs at 7 and 14 days post-surgery (scale bar: 100 μm).

To verify whether the PGA-PL hydrogel had any toxic or adverse effects on the major organs of rats, histopathological examinations were performed on rats 7 and 14 days post-surgery. As shown in Figure 5, compared with the control group, the hearts, livers, spleens, lungs, and kidneys of rats in the PGA-PL group exhibited intact tissue structure and normal cellular morphology following HE staining, with no obvious pathological changes observed. Specifically: In the heart, myocardial fibers were neatly arranged with distinct striations, and the interstitium showed no edema, hemorrhage, or inflammatory cell infiltration; in the liver, the structure of the hepatic lobules was clear, hepatocyte cords were neatly arranged, and no steatosis, necrosis, or abnormal hyperplasia was observed; the portal area showed no signs of inflammatory cell aggregation or fibrosis. In the spleen, the structures of the white and red pulp were distinct with a normal ratio, and lymphocytes were evenly distributed; no hemorrhage, necrosis, or abnormal hyperplasia was observed. In the lungs, alveolar structures were intact, with no thickening of the septa, and no inflammatory exudate or fibrosis was observed around the bronchi or blood vessels. In the kidneys, the structures of the glomeruli and renal tubules were clear, with no signs of congestion, edema, inflammatory infiltration, or interstitial fibrosis.

In summary, the experimental results indicate that this material did not cause significant toxic damage to the major organs of rats during the experimental period and demonstrates good tissue compatibility and in vivo safety.

### 2.7. PGA-PL Inhibits Inflammatory Responses

**Figure 6:**
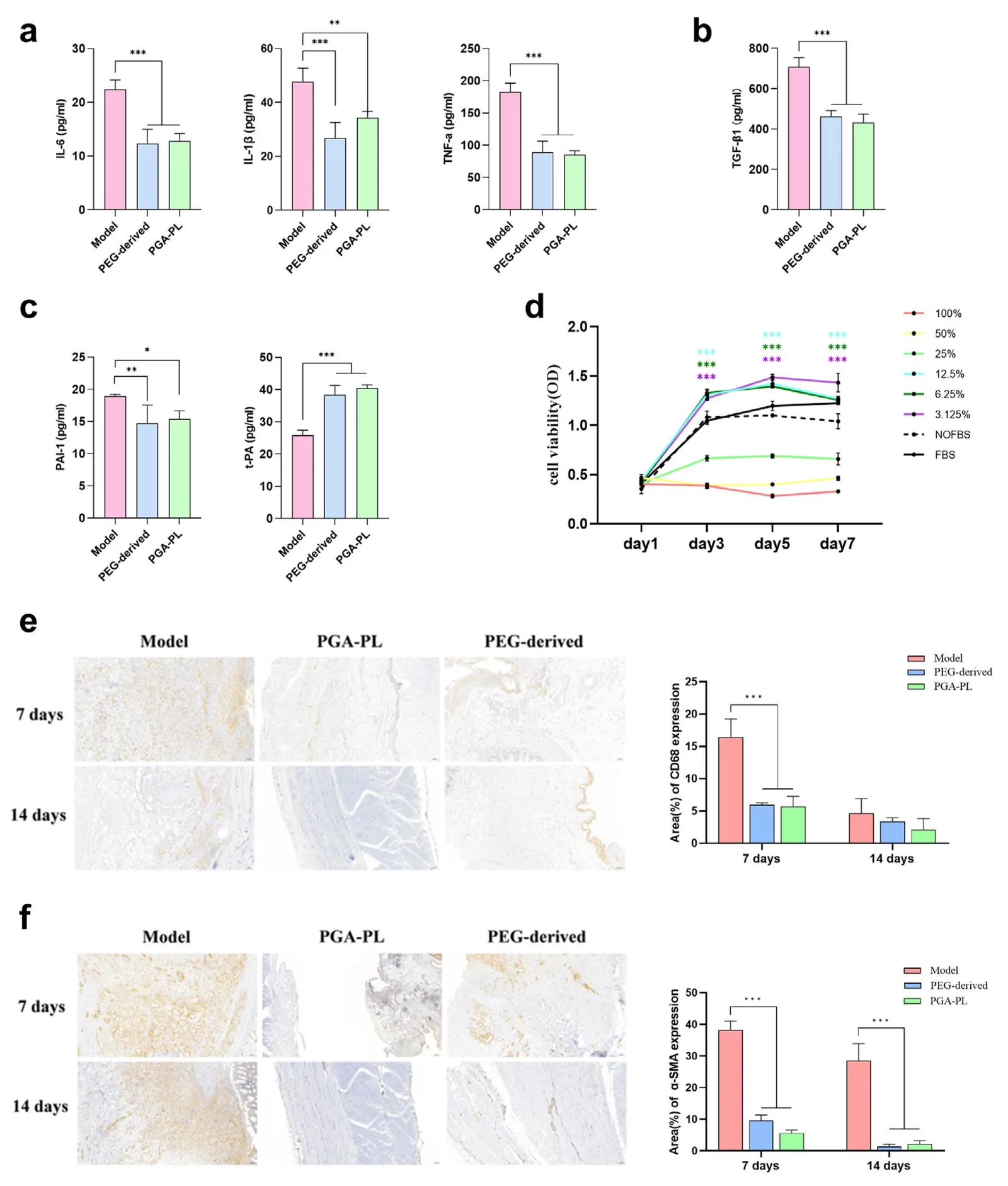
(**a**) Expression levels of inflammatory factors IL-6, IL-1β, and TNF-α in postoperative rat serum (*n* = 5); (**b**) Expression of TGF-β1 in the serum of postoperative rats (*n* = 5); (**c**) Postoperative serum concentrations of PAI-1 and t-PA in rats (*n* = 5); (**d**) Cell proliferation absorbance of PGA-PL extracts at different dilution ratios (*n* = 6); (**e**) Expression of CD68 in the adhesion band after application of the hydrogel in the rat cecum–abdominal wall adhesion model (*n* = 5). (**f**) Expression of α-SMA in the adhesion band. (*n* = 5) * *p* < 0.05, ** *p* < 0.01, *** *p* < 0.001.

During peritoneal adhesion formation, the injured site triggers a series of acute inflammatory responses: increased vascular permeability, massive recruitment of inflammatory cells including neutrophils and macrophages to the damaged area, and release of multiple inflammatory mediators such as IL-6, IL-1β, and TNF-α [5]. These factors not only drive the inflammatory cascade but also further promote fibrin exudation and deposition, and activate fibroblasts to progress toward fibrosis. In this study, we used ELISA to quantitatively detect the levels of IL-6, IL-1β, and TNF-α in rat serum 14 days post-surgery across all groups to assess the systemic inflammatory state in the model, thereby determining whether PGA-PL hydrogel exerts its anti-inflammatory and anti-adhesion effects by regulating the expression of these key inflammatory factors. As shown in Figure 6a, in the cecum–abdominal wall defect model, compared with the control group, treatment with PGA-PL hydrogel significantly reduced the expression levels of all three inflammatory factors in serum, particularly IL-6 and TNF-α (*p* < 0.001), and these levels were comparable to those in the normal group.

CD68 is a specific surface marker of macrophages. In the early stages of adhesion formation, macrophages—as the primary inflammatory cells—aggregate at the site of peritoneal injury and release large amounts of inflammatory factors. As shown by the immunohistochemical staining in Figure 6e, extensive macrophage infiltration was observed in the model group on postoperative day 7. Following treatment with the PGA-PL hydrogel, the number of macrophages infiltrating the locally injured cecum and abdominal wall tissues was significantly reduced (*p* < 0.001), and this finding was comparable to that of the commercially available anti-adhesion hydrogel, PEG-derived.

### 2.8. PGA-PL Inhibits Peritoneal Fibrosis

The late inflammatory phase and the initiation of fibrosis are critical stages in peritoneal adhesions. Once activated, macrophages and other cells recruited to the injury site continuously release TGF-β1. As the most potent pro-fibrotic factor, TGF-β1 strongly drives the differentiation of fibroblasts into myofibroblasts and significantly promotes the synthesis and deposition of the extracellular matrix, thereby directly mediating the fundamental transition from early inflammatory exudation to the formation of permanent fibrotic adhesions in the later stages. By measuring postoperative serum TGF-β1 levels in rats, it is possible to directly assess the intensity of fibrosis activation. The results of immunohistochemical analysis are shown in Figure 6b. At 7 days post-surgery, TGF-β1 was highly expressed in the adhesion band. Following treatment with either of the two hydrogels, TGF-β1 expression in the locally damaged peritoneum was significantly downregulated (*p* < 0.001). Furthermore, the PGA-PL hydrogel demonstrated a stronger downregulatory effect than the commercially available PEG-derived hydrogel. These results indicate that the PGA-PL hydrogel can reduce fibroblast proliferation and inhibit the progression of adhesions.

α-SMA is a hallmark protein of myofibroblast activation. Myofibroblasts are key effector cells in tissue fibrosis; they arise from the transformation of mesothelial cells and fibroblasts and possess a strong capacity for contraction and secretion of the extracellular matrix. Their persistent presence and activation form the structural basis for the maturation, organization, and contracture of adhesions. Figure 6f directly indicates fibrosis activity and the maturity of adhesions by measuring the intensity of α-SMA expression via immunohistochemistry. In the model group, the adhesion bands showed strong positivity and high expression of α-SMA, suggesting extensive fibroblast infiltration and the formation of fibrous adhesions within the bands. Following treatment with PGA-PL hydrogel or a commercial hydrogel, α-SMA expression in the locally damaged peritoneum was significantly downregulated compared to the control group at both 7 and 14 days post-surgery (*p* < 0.001), indicating that the PGA-PL hydrogel can inhibit the fibrotic process in the locally damaged peritoneum.

### 2.9. PGA-PL Regulates the Balance of the Fibrinolytic System

Dysregulation of the fibrinolytic system is a key contributing factor to peritoneal adhesion formation. Whether the fibrin exuded at the injury site can be cleared in a timely manner determines whether the temporary fibrin matrix will develop into permanent adhesion. This process is mainly regulated by the dynamic equilibrium between tissue plasminogen activator (t-PA) and its specific endogenous inhibitor, plasminogen activator inhibitor-1 (PAI-1). Elevated PAI-1 level strongly inhibits t-PA activity, leading to impaired fibrinolytic function and persistence of the fibrin network, which provides a stable scaffold for fibroblast infiltration and collagen deposition, directly promoting adhesion consolidation [22,23]. As shown in Figure 6c, compared with the blank normal group, the model control group presented high PAI-1 expression and low t-PA expression. Although PGA-PL hydrogel treatment showed limited effect on downregulating PAI-1 expression, it significantly promoted the upregulation of t-PA (*p* < 0.001), restoring its level to a range comparable to the normal group. This result indicates that the hydrogel can modulate the expression levels of PAI-1 and t-PA to restore the homeostasis of the fibrinolytic system at the injured site.

### 2.10. PGA-PL Promotes Mesothelial Regeneration

The mesothelium is a smooth, moist, semipermeable membrane that lines the peritoneal cavity, protecting intra-abdominal organs from friction damage and preventing bacterial infection. As the major cell type of the peritoneum, mesothelial cells are the first to be affected when peritoneal injury occurs [24]. Accordingly, the functional status of mesothelial cells directly determines the outcome of peritoneal repair. The cell proliferation activity of HMrSV5 cells co-cultured with PGA-PL hydrogel extracts is shown in Figure 6d. On day 3, 5, and 7, the OD values of the three groups with extract dilution concentrations no higher than 12.5% were significantly higher than those of the control group (*p* < 0.001), and also higher than those of the positive control group. This result indicates that PGA-PL hydrogel has a significant pro-proliferative effect on peritoneal mesothelial cells.

## 3. Discussion and Conclusions

This study is the first to systematically investigate the anti-adhesion effect of PGA-PL composite hydrogel and reveal its potential associations with multiple pathways involved in adhesion formation.

The anti-adhesive efficacy of PGA-PL hydrogel stems not only from its excellent physical barrier properties but may also be associated with the intrinsic anti-inflammatory and antioxidant potentials of its two components, γ-PGA and ε-PL. Experimental results demonstrate that PGA-PL hydrogel is correlated with the systematic reduction in pro-inflammatory factors and decreased macrophage infiltration at injury sites. This is consistent with recent findings that ε-polylysine-based biomaterials can modulate macrophage polarization toward the M2-like phenotype and reduce reactive oxygen species levels, thereby mitigating inflammatory responses [25,26]. Notably, these molecular-level alterations may represent direct biological effects exerted by hydrogel components, or secondary outcomes resulting from mitigated tissue trauma and reduced adhesion formation. Further mechanistic investigations are required to clarify the exact causal relationship.

More importantly, we observed that PGA-PL hydrogel is linked to the regulation of core fibrotic pathways. It can significantly downregulate the expression of TGF-β1, a pivotal factor driving mesothelial-to-mesenchymal transition and excessive extracellular matrix deposition. The inhibitory effect of the hydrogel on TGF-β1 and its downstream effector protein α-SMA may interfere with key steps in adhesive tissue maturation; nevertheless, such an effect may also be a secondary manifestation following the alleviation of inflammation and adhesions, rather than a direct targeting action of the hydrogel.

In summary, the composite PGA-PL hydrogel is a novel injectable anti-adhesive material featuring facile preparation and favorable biocompatibility. It effectively prevents and treats postoperative peritoneal adhesions via multiple potential mechanisms, including physical isolation as well as effects related to anti-inflammation, fibrinolysis regulation, anti-fibrosis and tissue repair promotion. Further studies are still needed to establish the direct causal relationship between the hydrogel and the observed molecular and cellular changes.

## 4. Materials and Methods

### 4.1. Instruments and Materials

#### 4.1.1. Instruments

The UV-Vis spectrophotometer (U-3900; Hitachi) was purchased from Hitachi, Ltd. (Tokyo, Japan). The shaking incubator (ZS-BPM) was obtained from Zhejiang Huayuan Instrument Co., Ltd. (Hangzhou, China). The inverted fluorescence microscope (CKX53; Olympus) was purchased from Olympus Corporation (Tokyo, Japan). The automated cell fluorescence analyzer (Mira FL) was provided by Shanghai Ruiyu Biotechnology Co., Ltd. (Shanghai, China). The multifunctional microplate reader (SpectraMax M5; Molecular Devices) was purchased from Molecular Devices, Inc. (San Jose, CA, USA). The magnetic stirrer (HJ-6A) was obtained from Changzhou Ronghua Instrument Manufacturing Co., Ltd. (Changzhou, Jiangsu, China). The centrifuge (L550) was purchased from Hunan Xiangyi Instrument Co., Ltd. (Changsha, China).

#### 4.1.2. Materials

Sodium γ-polyglutamate (γ-PGA) was provided by Furada Co., Ltd. (Jinan, China). ε-polylysine hydrochloride (ε-PL) and N-hydroxysuccinimide (NHS) were purchased from Aladdin Biochemical Technology Co., Ltd. (Shanghai, China). 1-ethyl-3-(3-dimethylaminopropyl) carbodiimide hydrochloride (EDC) was obtained from Damas-beta (Shanghai, China), and 4-morpholinoethanesulfonic acid (MES) was purchased from Sigma-Aldrich (St. Louis, MO, USA). The DPPH radical scavenging assay kit and bovine serum albumin (BSA) were bought from Solabio Technology Co., Ltd. (Beijing, China), and fibrinogen (Fbg) was purchased from Biosharp Life Sciences (Hefei, China).

Rabbit anti-TGF-β1 antibody and rabbit anti-CD68 antibody were purchased from Abcam (Cambridge, UK). Mouse anti-α-SMA antibody was obtained from Thermo Fisher Scientific (Waltham, MA, USA). The IL-6 ELISA kit, IL-1β ELISA kit, TNF-α ELISA kit, TGF-β1 ELISA kit, PAI-1 ELISA kit, and t-PA ELISA kit were all purchased from Youpin Biotechnology Co., Ltd. (Shenzhen, China). HRP-labeled goat anti-rabbit IgG and HRP-labeled goat anti-mouse IgG were provided by Servicebio Technology Co., Ltd. (Wuhan, China).

Sprague–Dawley (SD) rats were supplied by the Animal Resource Center of the National Institutes for Food and Drug Control (Beijing, China). All animal experiments were approved by the Institutional Animal Care and Use Committee, with the ethics approval number Zhongjian Dong (Fu) No. 2025 (B) 073.

### 4.2. Preparation of PGA-PL Hydrogels

The PGA-PL hydrogel for anti-adhesion models was prepared via an EDC/NHS-mediated crosslinking reaction. Specifically, an appropriate amount of γ-PGA was stirred and dissolved in MES buffer (pH 3.61) to obtain Solution A containing γ-PGA. Meanwhile, ε-PL was dissolved in the same buffer to prepare Solution B. Solution B was then added dropwise to stirred Solution A, and the resulting homogeneous mixture was designated as Solution C. NHS was added to Solution C, and the mixed system was incubated at low temperature for 0.5 h. Finally, EDC was introduced into the reaction system to trigger gelation, yielding the PGA-PL hydrogel (the molar ratio of PGA, PL, EDC and NHS was 10:2:2.5:2.5).

Structural characterization and physicochemical property studies of the PGA-PL hydrogel were completed in a previous phase of this experiment; for details, see “Li, J. et al. Gels 11, 226 (2025)” [27].

### 4.3. Determination of Antioxidant Activity

The DPPH radical, a highly stable nitrogen-centered free radical, is widely used as a key indicator for evaluating the antioxidant capacity of samples. The DPPH radical carries an unpaired electron; its ethanolic solution appears purple and has a characteristic absorption peak at 515 nm. In the presence of antioxidants, DPPH radicals are scavenged and neutralized, leading to color fading of the solution and a decrease in absorbance at 515 nm. Within a certain range, the change in absorbance is directly proportional to the degree of radical scavenging. In this study, the antioxidant performance of hydrogels was evaluated by measuring their scavenging efficiency against DPPH radicals.

The experiment was divided into three groups: positive control group, PGA-PL hydrogel groups (including three formulations with monomer mass ratios of 10:0.2, 10:0.1, and 10:0.7), and commercial PEG-derived hydrogel group. Blank tubes, sample tubes, control tubes, and positive control tubes were prepared according to the kit instructions. Briefly, 200 μL of the test hydrogel sample was added to each corresponding tube, and 10 μL of vitamin C solution was added to the positive control group. All Eppendorf tubes were vortexed for thorough mixing, and then incubated at room temperature in the dark for 30 min. After incubation, the absorbance of the supernatant from each group was measured at 515 nm using a UV-Vis spectrophotometer (Hitachi, Ltd., Tokyo, Japan) [28].

The radical scavenging rate was calculated using the following formula: D (%) = [(Ab − An)/Ab] × 100%, where Ab represents the absorbance value of the blank well, and An represents the absorbance value of the control or experimental group. Each group was set with 4 replicate wells.

### 4.4. Anti-Fouling Performance Testing

Non-specific protein adsorption and cell adhesion are inevitable challenges during the implantation of hydrogels into the peritoneal cavity, and excessive collagen, fibroblasts, and macrophages are direct contributors to the formation of adhesions. Therefore, a comprehensive evaluation of the anti-fouling capabilities of the PGA-PL hydrogel is crucial.

For the non-specific protein adsorption assay, bovine serum albumin (BSA) and fibrinogen (Fbg) were selected as non-specific proteins. An appropriate volume of PBS was added to PGA-PL hydrogels and commercial PEG-derived hydrogels in a 24-well plate. After shaking on a shaker for several minutes, the excess liquid was discarded. This process was repeated three to four times until the hydrogels reached swelling equilibrium. Add 1 mL of protein solution (2 mg/mL) to each well and incubate with shaking at 37 °C for 2 h. Subsequently, aspirate the excess protein solution and rinse the hydrogel surface with 0.9% NaCl solution, shaking for 3 min, repeating this process three times to remove loosely adsorbed proteins from the hydrogel wells. Add 500 μL of 1% sodium dodecyl sulfate (SDS) to each well and incubate for 1 h at 37 °C on a 200 rpm shaker to separate proteins tightly adsorbed to the hydrogel surface; collect the separated proteins in a centrifuge tube. After incubation, determine the concentration of residual protein using a BCA protein assay kit (Solarbio, Beijing, China). Polystyrene for tissue culture (TCP) was used as the control group, and a hydrogel blank group was established, with four replicate wells in each group [29].

L929 cells and RAW264.7 cells were selected for the cell adhesion assay. Cells were seeded at a density of 5 × 10^4^ cells/well onto PGA-PL hydrogels, commercially available PEG-derived hydrogels, and the control group of tissue culture polystyrene (TCPs) in a 24-well plate. The plates were incubated in a cell culture incubator for 24 h. After washing twice with PBS, the adhesion of L929 and RAW264.7 cells was observed using an inverted microscope (Olympus, Tokyo, Japan). Following observation, 0.4 mL of trypsin was added to each well in each group and incubated for 3 min, after which 0.8 mL of high-glucose DMEM complete medium was immediately added to terminate digestion. Cells in each well were counted using an automatic cell counter (Ruiyu Biotechnology Co., Ltd., Shanghai, China). Each group consisted of 4 replicate wells; L929 cells and RAW264.7 cells were selected for the cell adhesion assay. Cells were seeded at a density of 5 × 10^4^ cells/well onto PGA-PL hydrogels, commercially available PEG-derived hydrogels, and the control group of tissue culture polystyrene (TCPs) in a 24-well plate. The plates were incubated in a cell culture incubator for 24 h. After washing twice with PBS, the adhesion of L929 and RAW264.7 cells was observed using an inverted microscope. Following observation, 0.4 mL of trypsin was added to each well in each group and incubated for 3 min, after which 0.8 mL of high-glucose DMEM complete medium was immediately added to terminate digestion. Cells in each well were counted using an automatic cell counter. Each group consisted of 4 replicate wells [29].

### 4.5. Animal Experiments

#### 4.5.1. Establishment of a Rat Cecum–Peritoneum Adhesion Model

Forty healthy SD rats (half male and half female) weighing 200 ± 20 g were selected for the experiment. After one week of acclimatization to the laboratory environment, the rats were randomly divided into four groups: blank control group, model control group, PGA-PL hydrogel treatment group, and commercial PEG-derived anti-adhesion hydrogel treatment group, with 10 rats in each group. All rats were fasted for 24 h before surgery. After brief inhalation anesthesia with isoflurane, intraperitoneal anesthesia was administered. The lower abdomen was shaved and disinfected with povidone-iodine. A 5 cm midline incision was made through the skin and abdominal wall to expose the abdominal cavity. The cecum was gently exposed, and a sterile surgical gauze pad was held with surgical forceps to abrade the serosal surface from the base of the cecum upward, covering an area of 1 × 2 cm^2^ with approximately 30 scrapes until punctate bleeding appeared. Simultaneously, the lateral abdominal wall on the same side was exposed and abraded 30 times using the same method. A 1 mL syringe was used to apply 0.2 mL of anhydrous ethanol onto the abraded cecal surface, which was left in place for 30 s to induce chemical injury. Then, the anhydrous ethanol was absorbed with sterile gauze. The blank control group received no traumatic treatment. The model control group received 0.5 mL of normal saline applied to the abdominal wall and cecal wound surfaces using a needle-free syringe. The PGA-PL group was treated with 0.5 mL of PGA-PL hydrogel via topical application and local injection. The commercial hydrogel group received 0.5 mL of the corresponding PEG-derived hydrogel in the same manner. After air exposure for several minutes, the abraded surfaces of the cecum and lateral abdominal wall were brought into contact, and the abdominal cavity was closed layer by layer. The abdominal wall and skin were sutured with 5-0 and 3-0 sutures, respectively [30].

On postoperative days 7 and 14, 5 animals were randomly selected from each group and anesthetized. The abdominal skin was incised via a “U”-shaped incision to observe the adhesion between the abdominal wall and the cecum, and the findings were scored according to the Diamond scoring system in Table 1.

#### 4.5.2. Sample Collection

Tissue samples: Anesthetize the rat and make a “U”-shaped incision in the abdominal skin; avoid using previous surgical incisions to ensure tissue integrity. After exposing the abdominal cavity, observe and record the presence of adhesions within the cavity. For rats with adhesions between the intestinal wall and the abdominal wall, the adherent areas should be precisely excised. If no adhesions are present, remove the damaged cecum and abdominal wall tissue. When flushing the intestinal contents, take care not to dislodge any adherent tissue. After collecting the tissue samples, process them in two ways: one portion is immersed in a pathology cup containing 4% paraformaldehyde and left at room temperature, while the other portion is stored at −80 °C for cryopreservation.

Serum Samples: After exposing the abdominal cavity, blood was immediately collected from the abdominal aorta. The blood collection tubes were allowed to stand at room temperature for 30 min, then centrifuged at 3000 rpm for 10 min to collect the supernatant. The supernatant was centrifuged again at 3000 rpm for 5 min to obtain the final serum sample, which was stored at −80 °C until further analysis.

Organ Harvesting: Hearts, livers, spleens, lungs, and kidneys were harvested from rats in the target groups. Lungs were perfused with 4% paraformaldehyde to ensure complete filling. All organs were immersed in pathology cups containing 4% paraformaldehyde and stored at room temperature.

### 4.6. Histopathological Analysis

#### 4.6.1. H&E Staining

Tissue samples were harvested to a thickness of approximately 3 mm. The samples were dehydrated in a gradient of ethanol (70%, 80%, 95%, and 100%) for 90 min each, followed by 20 min each in xylene I and xylene II, and then embedded in paraffin wax for 15 min in each of two tanks. Embed in paraffin and section into slides approximately 3 μm thick.

Deparaffinization: Soak in xylene three times, 8 min each. Rehydration: Soak in anhydrous ethanol twice, 8 min each, followed by 8 min soaks in 90%, 80%, and 60% ethanol. Hematoxylin staining for 4 min, followed by rinsing under running water.

Differentiation with hydrochloric acid and alcohol for 2–3 s, followed by rinsing under running water; 0.5% ammonia solution for 20 s, followed by rinsing under running water, then microscopic examination. 0.5% eosin staining for 1 min; differentiation with 80% and 90% ethanol for 3–5 s each; 95% ethanol for 5 min; three immersions in anhydrous ethanol, 5 min each; two immersions in xylene, 5 min each. Mount with neutral resin, examine under a light microscope (Sunny Instruments Co., Ltd., Ningbo, China), and take microphotographs.

#### 4.6.2. Masson Staining

Paraffin sections were dewaxed to water, as described above. Stained with ferric hematoxylin solution for 10 min. Rinsed rapidly with water; acidified with acid solution, then rapidly decolorized; blued with ammonia solution; and thoroughly rinsed with tap water. Stained with acid red solution for 15 min. Decolorized with aqueous phosphomolybdic acid solution for 30 s. Immediately immersed in aniline blue solution for 2 min. Rinsed rapidly under running water. Dehydrated with anhydrous ethanol until clear, then mount.

### 4.7. Serological Testing

#### 4.7.1. Blood Biochemical Analysis

Take an appropriate amount of serum sample and measure aspartate aminotransferase (AST), alanine aminotransferase (ALT), alkaline phosphatase (ALP), urea (UREA), total protein (TP), albumin (ALB), glucose (GLU), creatinine (CRE), total cholesterol (T-CHO). This will evaluate the effects of the PGA-PL gel and the currently available commercial PEG-derived hydrogel on liver function, kidney function, blood lipids, blood glucose, and serum proteins in SD rats.

#### 4.7.2. ELISA

The experiment was conducted strictly according to the ELISA kit instructions (Youpin Biotechnology Co., Ltd., Shenzhen, China). For TGF-β1 measurement, the serum sample dilution factor was 2; for the remaining parameters, the dilution factor was 2.5. Immediately after the experiment, the microplate was placed in a microplate reader (Molecular Devices, Inc., San Jose, CA, USA) to measure the OD values at 450 nm for each well.

With the concentration of the calibration standard as the x-axis and the corresponding OD value as the y-axis, a four-parameter logistic curve was fitted to generate the standard curve equation. This was used to calculate the sample concentration, which was then multiplied by the dilution factor to obtain the final sample concentration.

### 4.8. Immunohistochemistry

Perform CD68, TGF-β1, and α-SMA immunohistochemical staining on sections of adherent tissue to quantitatively analyze macrophage infiltration and the expression of fibrosis-related proteins.

### 4.9. Cell Proliferation Assay

An appropriate amount of sterilized PGA-PL hydrogel was dissolved in RPMI 1640 medium containing 10% serum. The hydrogel was allowed to fully swell and extract in a 37 °C incubator for 24 h. The supernatant was then collected and filtered through a 0.22 μm filter to remove bacteria, yielding a 0.1 g/mL extract (100% concentration). This solution was serially diluted to prepare 100%, 50%, 25%, 12.5%, 6.25%, and 3.125% dilutions.

Seed HMrSV5 at 2 × 10^3^ cells/100 μL in a 96-well plate and cover the wells with RPMI 1640 medium containing 10% serum. After 24 h, change the medium and add the PGA-PL hydrogel extract solutions at the six concentration gradients for co-culture; the serum group served as the positive control, and the serum-free group as the negative control. After 1, 3, 5 and 7 days, the culture medium in the plates was discarded, and a CCK-8 solution prepared with serum-free medium at a 1:9 ratio was added. OD values were measured at 1, 3 and 7 days using a microplate reader, and cell viability was calculated.

### 4.10. Statistical Analysis

All experiments were independently repeated at least three times (*n* ≥ 3) and presented as mean ± standard deviation. Statistical significance between the results was determined by one-way ANOVA and Student’s *t*-test. *p* < 0.05 was considered to be statistically significant (* *p* < 0.05, ** *p* < 0.01, *** *p* < 0.001).

## Figures and Tables

**Table 1:** Diamond Adhesion Score Chart.

Characteristics		Adhesion Score
Degrees	None	0
	≤25%	1
	≤50%	2
	≤75%	3
	>75%	4
Types	None	0
	Thin, transparent adhesions without blood vessels	1
	Semi-transparent or opaque adhesions without blood vessels	2
	Opaque adhesions with distinct small blood vessels	3
	Opaque adhesions with prominent large blood vessels	4
Tenacity	None	0
	Adhesions separate easily	1
	Adhesions separate upon traction	2
	Adhesions require sharp dissection	3

## Data Availability

The data presented in this study are available in this article.

## Appendix A

### Appendix A.2. Effect of PGA-PL Hydrogel in a Cecum-Peritoneum Adhesion Model

**Table A1:** The adhesion results for rats in each group.

Time	Group	Results
7 days	Model	Rats 1 and 2 exhibited moderate adhesions between the cecum and the abdominal wall, covering <25% of the area.
		Rat 3 exhibited tight adhesions between the cecum and the abdominal wall, covering >25% of the area. In all three cases, a white, opaque adhesion band containing small red blood vessels was observed forming between the cecum and the peritesticular fat tissue, requiring sharp dissection.
		Rat 4 exhibited tight adhesion between the cecum and the abdominal wall, covering an area >25%, and formed a relatively transparent adhesion band with the tissues surrounding the bladder; this band was thin and contained distinct small blood vessels, requiring sharp dissection.
		Rat 5 exhibited adhesion between the cecum and the abdominal wall covering an area <25%, requiring sharp dissection.
	PEG-derived	1 specimen exhibited moderate adhesions, while 4 had no adhesions: Although the cecum of specimen No. 5 was not adherent to the abdominal wall, it formed a transparent adhesion band with other parts of the intestine, containing small blood vessels; the adhesion could be separated by traction.
	PGA-PL	4 animals showed no adhesions and 1 showed mild adhesions: the cecum No. 1 formed a single adhesion band with the abdominal wall, covering an area of less than 25%; the band was translucent, lacked blood vessels, and could be separated by traction.
14 days	Model	Cecums No. 6 and No. 7 were tightly adherent to the abdominal wall, covering >25% of the surface.
		Cecum No. 8 showed no obvious adhesion to the wound surface. In all three cases, the cecum formed a white, opaque adhesion band containing small red blood vessels with the peritesticular fat tissue, requiring sharp dissection.
		The ceca of specimens 9 and 10 developed extensive adhesions to the lower and lateral abdominal walls, respectively, with an adhesion area of >50% for specimen 9 and >25% for specimen 10; both contained distinct small blood vessels and required sharp dissection.
	PEG-derived	one specimen exhibited severe adhesions, three had mild adhesions, and one had no adhesions: only the cecum of specimen No. 8 was tightly adherent to the abdominal wall, with an adhesion area >25%. Additionally, the cecum and the fat tissue surrounding the testis formed two white, opaque adhesion bands containing large red blood vessels, requiring sharp dissection. In specimens 6 and 7, the adhesions formed between the cecum and the peritesticular fat tissue contained small red blood vessels and were easily separated; in specimen 9, the adhesion was avascular and could be separated by traction.
	PGA-PL	none of the 5 ceca showed adhesions to the abdominal wall. Only specimen 9 exhibited a single adhesion band between the lower abdominal wall and the surrounding abdominal tissue, covering an area of less than 25%; this band was translucent, avascular, and could be separated by traction.
